# A Novel BrainHealth Index Prototype Improved by Telehealth-Delivered Training During COVID-19

**DOI:** 10.3389/fpubh.2021.641754

**Published:** 2021-03-16

**Authors:** Sandra Bond Chapman, Julie M. Fratantoni, Ian H. Robertson, Mark D'Esposito, Geoffrey S. F. Ling, Jennifer Zientz, Stacy Vernon, Erin Venza, Lori G. Cook, Aaron Tate, Jeffrey S. Spence

**Affiliations:** ^1^Center for BrainHealth^®^, School of Behavioral and Brain Sciences, The University of Texas at Dallas, Dallas, TX, United States; ^2^Institute of Neuroscience, Global Brain Health Institute, Trinity College Dublin, Dublin, Ireland; ^3^Department of Molecular and Cell Biology, Helen Wills Neuroscience Institute, University of California, Berkeley, Berkeley, CA, United States; ^4^Department of Neurology and Neuroscience, School of Medicine, Johns Hopkins University, Baltimore, MD, United States

**Keywords:** brain health, digital health, mental health, neuroplasticity, pandemic, personalized care, prevention, resilience

## Abstract

**Introduction:** Brain health is neglected in public health, receiving attention after something goes wrong. Neuroplasticity research illustrates that preventive steps strengthen the brain's component systems; however, this information is not widely known. Actionable steps are needed to scale proven population-level interventions.

**Objectives:** This pilot tested two main objectives: (1) the feasibility/ease of use of an online platform to measure brain health, deliver training, and offer virtual coaching to healthy adults and (2) to develop a data driven index of brain health. Methods: 180 participants, ages 18–87, enrolled in this 12-week pilot. Participants took a BrainHealth Index™ (BHI), a composite of assessments encompassing cognition, well-being, daily-life and social, pre-post training. Participants engaged in online training with three coaching sessions. We assessed changes in BHI, effects of training utilization and demographics, contributions of sub-domain measures to the BHI and development of a factor analytic structure of latent BrainHealth constructs.

**Results:** The results indicated that 75% of participants showed at least a 5-point gain on their BHI which did not depend on age, education, or gender. The contribution to these gains were from all sub-domains, including stress, anxiety and resilience, even though training focused largely on cognition. Some individuals improved due to increased resilience and decreased anxiety, whereas others improved due to increased innovation and social engagement. Larger gains depended on module utilization, especially strategy training. An exploratory factor analytic solution to the correlation matrix of online assessments identified three latent constructs.

**Discussion/Conclusion:** This pilot study demonstrated the efficacy of an online platform to assess changes on a composite BrainHealth Index and efficacy in delivering training modules and coaching. We found that adults, college age to late life, were motivated to learn about their brain and engage in virtual-training with coaching to improve their brain health. This effort intends to scale up to thousands, thus the pilot data, tested by an impending imaging pilot, will be utilized in ongoing machine learning (ML) algorithms to develop a precision brain health model. This pilot is a first step in scaling evidence-based brain health protocols to reach individuals and positively affect public health globally.

## Introduction

Public health policies can profoundly benefit people at all levels of society to flourish around the world (1). There is however, a notable void in policies for the health of the most crucial organ in the human body—the brain. This absence is starkly highlighted by the COVID pandemic where rapidly deteriorating mental clarity and psychological well-being (2) have accompanied sudden adverse changes in economic markets, employment, and social systems. These detrimental effects are magnified by health, social, and economic disparities.

### Brain Health Is a More Policy-Ready Concept Than Mental Health

As a concept, the term “mental health” is widely used, but is a rather limited term focusing narrowly on emotional well-being. To date, the term mental health fails to capture broad aspects of the “mind” and the interdependencies of other mental capabilities and dimensions. The term “brain health” is less familiar, but, we argue, is more useful for public health policy action, for a number of reasons. First, the term mental health does not address cognitive abilities—or “cognitive capital,” i.e., the summated cognitive capacities of a population. This is crucially important because greater cognitive capital has been shown to predict higher levels of health generally, both physical and mental [e.g., (3)]. The cognitive capital of a country is also crucial for its economic prosperity because it enables populations to pivot more adeptly and adaptively in the face of economic shocks, rapid technological change, and environmental challenges (4, 5). An absence or reduction of such a flexible mindset leads to economic and social deterioration that will worsen mental and physical well-being (6).

A second reason that brain health is more policy-ready as a concept than mental health is that it encompasses what is predicted to be one of the greatest health challenges facing the world, one that particularly impacts low and middle income countries (7). This health challenge is the spiraling prevalence of dementia linked to the accelerating proportion of older people in all countries in the world, secondary to impressive lengthening of life expectancy (8). Additionally, impaired well-being is associated with dementia, but is often ignored in treatment considerations as the interrelationships are disregarded (9). Mental health as a concept does not afford policy implications for this enormous health challenge that, if unchecked, will swamp the resources and finances of health providers and services. Brain health, on the other hand, is “oven-ready” as a concept to be tested, with clear implications for policy, some of which we will describe in this paper.

Thirdly, the concept of brain health has positive connotations that avoid a certain stigma that attaches to mental health brought on due to the large focus on diagnosing deficits rather than considering individual potential. Brain health, on the other hand, organizes around capacity-building principles of neuroplasticity. Therefore, it affords vast opportunities for policymakers to advance population-scale interventions to millions of people, which could make headway in mitigating fear of being stigmatized, and to replace such concerns with individual empowerment. Brain health offers a positive message that is similar to successful heart-healthy interventions such as those advocating the value of aerobic exercise (10, 11). In sum, the semantics of mental health, as a concept, do not extend plausible multifaceted pathways to motivate desirable behavior change.

Fourthly, mental health has a confusing definition, because it combines “absence of mental illness” and imprecisely-defined ideas of emotional well-being (https://www.merriam-webster.com/dictionary/mental%20health). Such ambiguity makes mental health difficult to measure and hence fails to motivate behavioral change aimed at building it and intercepting issues before they become clinically significant deficits. Behavior change needs clear metrics to incentivize progress. Indeed, we manage what we measure. The absence of such metrics deters the sort of learning and behavior change we know to be essential to improve healthy habits (12). Yet the lack of a clear index of the health of the brain—the most important organ in the body, subserving all emotions, cognition, and behavior—is a major void for public health policy.

The concept of brain health lends itself to the creation of such an index, because it is a less confused term than mental health: It avoids the false dichotomies underpinning “mental health,” namely between mind and brain on the one hand, and emotion and cognition on the other. In this paper, we define, establish, and begin to validate a newly developed BrainHealth Index. We acknowledge that improvements in this index will require increased data over larger populations and longer time intervals of years. Nonetheless, this effort catelyzes that progress toward utilization of sophisticated data analytical methods, including, for example, machine learning.

Finally, brain health is a high-level category of health and actually incorporates the emotional and behavioral phenomena that comprise the concept of mental health. Brain health, in contrast to mental health, lends itself to wider public acceptability and greater opportunities for proactive, preventive interventions to potentially intercept concerns before they become clinically debilitating. Thus, it follows that population-scale policies will have tremendous impact if they improve not only the cognitive capacity and flexibility of individuals but also the well-being and social connectedness of millions of people, particularly in the context of global challenges such as a health pandemic, e.g., COVID19.

### Measuring Brain Health

Our existing health systems have a limited scope of brain health, viewing it through the lens of disorder and disease rather than optimal function. For example, the National Institutes of Health (NIH) does not have its own definition of brain health and instead, uses one provided by the National Institute on Aging (13). Yet identifying brain health with aging ignores the centrality of brain health to the well-being and physical health of all ages, and risks misclassifying the concept as a problem of aging. Furthermore, the primary old-age-focus of the NIA definition of brain health results in only four elements being included, namely cognitive health, motor function, emotional function, and tactile function. This NIA definition of brain health is limited in the breadth and scope of what the brain is capable of and responsible for achieving. Specifically, the NIA definition leaves out components of social interaction (social support, social engagement, compassion), daily life (nutrition, exercise, mindset, sleep, responsibilities), and well-being (resilience, mood, quality of life). The fact that brain health is multifaceted and complex dictates a more integrated approach, rather than the current fragmented, siloed approach where domains are either not assessed at all or only as separate entities. Our preliminary BrainHealth Index encompasses all of these components—cognition, well-being, social interaction, and daily life. This is the first time these components have been combined to capture a single measure of the brain in an inter-related, holistic sense. There is a need to shift our existing thinking and definitions to include all of these domains in order to better care for the brain and understand the multiple pathways to strengthen overall brain health capacity. By integrating large-scale data across multiple domains and time intervals, we can evaluate, interpret, and perhaps even predict how components interact to achieve maintained, increased, or diminished brain health, at both group and individual levels. For example, emotions such as anxiety and depression affect cognitive function (14–18) while strong cognitive functions—particularly executive and attention processes—aid emotional regulation and build resilience (19, 20). Social isolation has negative effects on emotional well-being and cognitive function (21, 22). Sleep (23), diet (24), and physical activity (25) all mutually interact and affect these other emotional, cognitive, and social functions (26–28). Furthermore, all of these different processes both depend on, and influence the structure and function of brain networks and systems (29, 30). Brain health depends on the complex, interwoven interactions of these multifaceted processes (cognition, well-being, social, life habits/responsibilities) and therefore should not be addressed independently of each other. For this reason, a composite brain health measure is needed. As stated above, this study represents the first attempt to create such a holistic composite, which we label the BrainHealth Index, deliverable on an online platform.

### Scalable, Policy-Relevant Brain Health Interventions

To our knowledge, there have been no scalable interventions developed and tested to increase synergistically the multifaceted aspects of brain health across all age groups in a population. Moreover, definitely none exists that is delivered entirely remotely through a telehealth platform. The most prominent and successful attempt at a scalable multi-faceted intervention was directed at older individuals at risk of dementia, known as the Finnish “FINGER” trial. The FINGER trial showed how a multi-modal intervention delivering vascular risk monitoring, cognitive training, and dietary and exercise advice, could significantly improve cognition (27). This important study has seeded a worldwide network of similar trials (see an overview at https://alz.org/wwfingers/overview.asp).

The present intervention protocols differs in two main ways from the FINGER intervention. First, this pilot study targets all adults, ranging in age from 18 to 90, with the aim to test group and individual ability to improve brain health for a range of outcomes including mental well-being, innovative thinking, work performance, and social connection at one of the most difficult economic times in our history. Additionally, the current effort lays the foundation to assess effectiveness in reducing, postponing, or halting dementia risk as individuals are followed over time from health to diagnosis. Secondly, and crucially in the context of the COVID-19 pandemic, the current pilot was delivered entirely remotely which is a key factor in making such efforts scalable at a global level. The FINGER protocol is labor-intensive and, in contrast to the very low cost of a remote program, very expensive.

Our work complements and builds on decades of positive findings of the possibility of enhancing cognitive and emotional function using verbal and/or procedural protocols ranging from teaching strategy aimed at improving efficiency of frontal-lobe-based executive networks (31–35) to enhancing mood and reducing anxiety through methods such as cognitive behavior therapy (35). The ability to scale such methods using web-based technology has led to their now being available entirely remotely to millions of users across the globe. Most online offerings do not have strong published evidence of their efficacy. One exception to this are the BrainHQ (www.brainhq.com) online training programs that have shown positive cognitive effects in a range of populations across many studies (36–42). One such study showed impressive gains in certain cognitive processes of older adults with real-life benefits maintained up to 10 years later with just a few booster sessions (39, 43). Their work has also shown to be associated with lowering driving accidents in older adults, and improvements in daily life independence (36, 38, 39). In larger, follow-up interventions, our goal is to include a range of evidence-based training methods, such as BrainHQ, selecting those with strong empirical support. We aim to test the extent to which participants can achieve gains on the holistic BrainHealth Index as we expand the study to larger enrollment with 10-year or longer follow-up in the BrainHealth Project.

Today more than ever, we need a global brain health strategy translatable across the lifespan (5). Fortunately, heart healthy practices, established by decades of observational and more recently interventional studies (44), lay out a roadmap for how to increase public awareness and health practices. Over the past five decades, the Framingham study has delivered tremendous knowledge regarding preventive lifestyle habits, annual objective measurements, and medical interventions when necessary (45, 46). The good news is that people everywhere have access to: (a) clear indices of their heart health and (b) science-driven information on what they need to do to improve their heart health (47).

### Pilot Study Goals

The present study offers professional-quality video-based interventions aimed at teaching tactical cognitive strategies previously shown to improve brain health, including stress management and cognitive strategy training. Additional informational videos on diet, exercise, and sleep hygiene were provided. Remotely-delivered, individualized coaching sessions with a trained coach helped participants understand and integrate this information. Our prior work has shown that cognitive strategy training can generalize beyond the trained domains to neural signatures underpinning brain health, such as increased brain blood flow, connectivity, and cortical thickness, with the brain changes linked to improved complex cognition, psychological well-being, real life functions, and social adeptness (31, 32, 48–52).

We want to acknowledge at the outset, that this pilot study is the first element of a planned three-phase BrainHealth project (https://brainhealth.utdallas.edu/programs/the-brainhealth-project/). The first major goal of the present pilot study was to test an online system for: (1) remote delivery and collection of assessments; (2) remote delivery of a set of training modules; and (3) the feasibility of virtual coaching sessions with individual participants. The second major goal of this pilot study was to develop an exploratory factor model of a BrainHealth Index derived from the assessments in the domains of cognition, well-being, real life, and social interaction.

## Methods

This study was performed in accordance with the standards provided by The University of Texas at Dallas IRB. All participants were informed about the study protocol before obtaining written informed consent.

### Recruitment

Participants were recruited for the study through word of mouth and email advertising. As the primary aim was to study the brain in health we recruited generally healthy adults. Participants completed an online screening form to determine if they qualified for the study. Inclusion criteria consisted of being: 18 years or older, able to access the internet (including access to a computer/smartphone/tablet), and being a proficient English speaker. Potential participants were excluded for any of the following reasons: being under age 18, having a diagnosed neurological disorder, diagnosed psychotic disorder or uncontrolled psychiatric disorder, history of brain injury, or any uncontrolled health issues. It is important to note participants were not excluded for general health risk factors (ex: obesity, diabetes, and autoimmune conditions).

**Figure 1:**
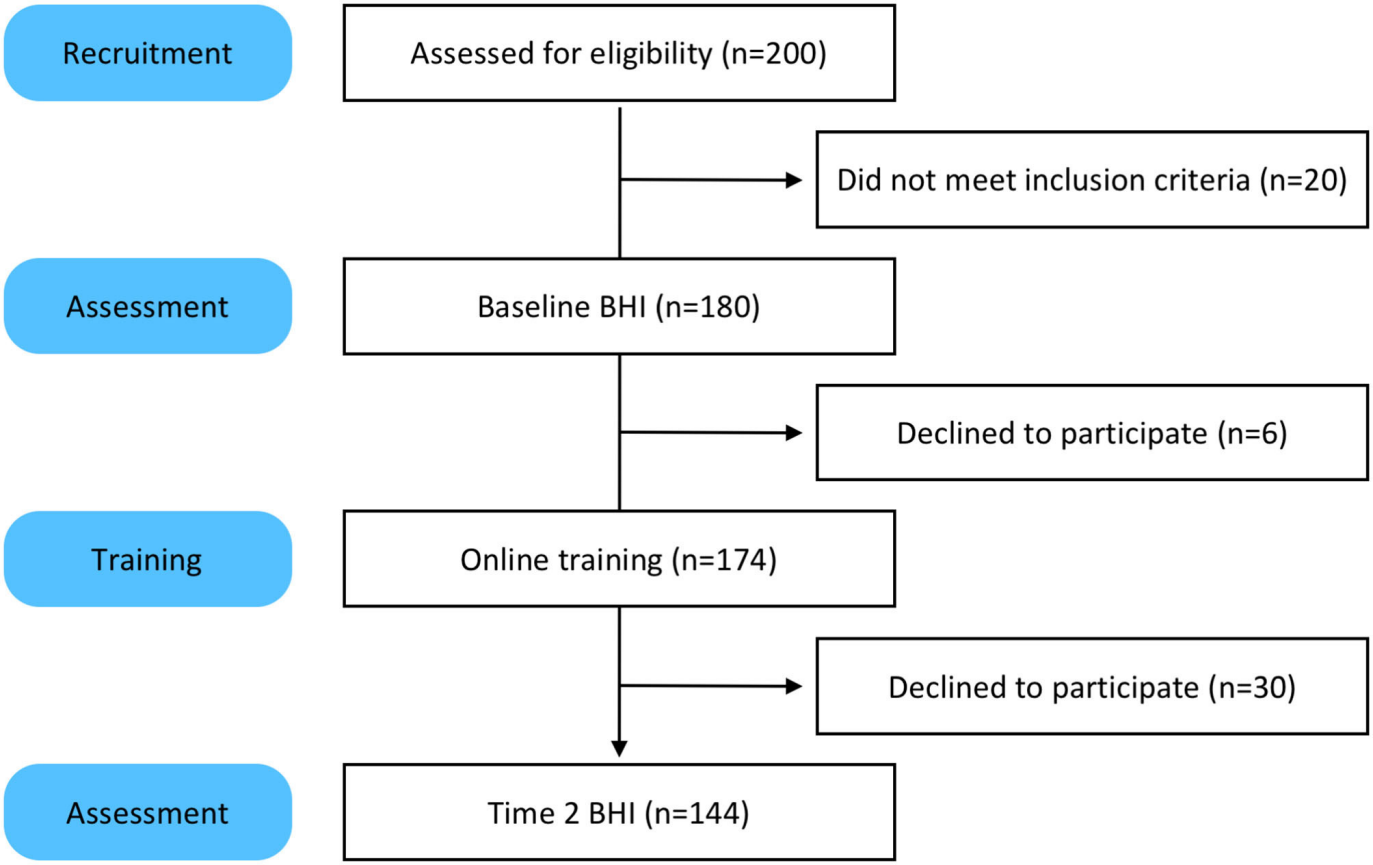
Participant recruitment and retention.

Two hundred people took the screener and 180 qualified for the study (Figure 1). One hundred eighty participants completed the baseline assessments for the BHI. Of those 180, 174 engaged in the online training and coaching. The six participants who did not engage in training were unresponsive to email prompts and reminders. One hundred and forty four participants took the Time 2 BHI. The 30 participants who did not complete the Time 2 BHI were sent reminders via email and phone. Most reported they were too busy and did not have time to take the assessments.

### Study Protocol

**Figure 2:**
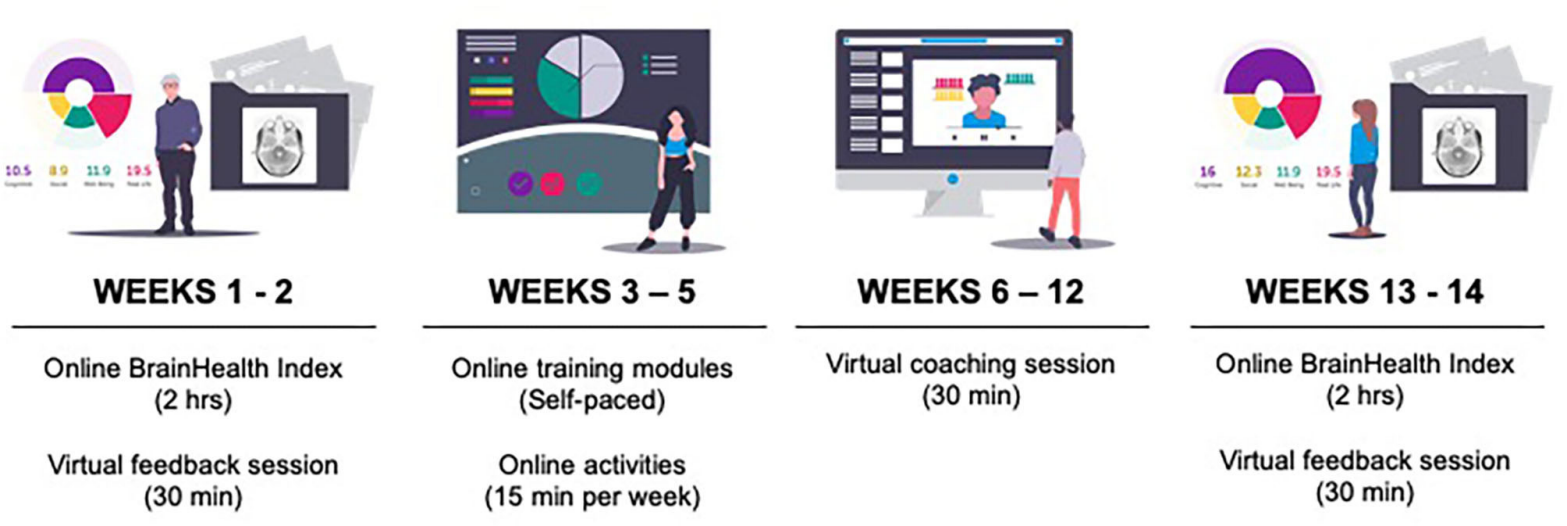
Study timeline.

This pilot study consisted of online assessments, coaching, and training over a period of 12 weeks (Figure 2).

First, participants completed the online assessments and then received their BrainHealth Index score. Week 2, participants had their first coaching call with a BrainHealth coach to discuss their BrainHealth Index results and get personalized recommendations for interacting with the online training based on those results. The BrainHealth coaches were all masters-degree level clinicians who were well-versed in the implementation of the content found in the training modules. Weeks 3–5, participants were directed to complete self-paced online training modules (described below). Week 6 was a halfway-point coaching call intended to motivate participants to continue implementing what they learned in training into real-life. Weeks 7–10, participants continued working through training modules. Week 11, participants took the assessments for the BrainHealth Index a second time. Week 12, participants had their third coaching call where they were able to learn about and compare the changes in their BrainHealth Index score.

### Assessments

Participants completed a series of online assessments that tapped into our four critical hypothesized domains of brain health: (1) cognition, (2) well-being, (3) social interaction, and (4) daily life (Table 1).

**Table 1:** List of online assessments.

**Domain**	**Measure**	**References**	**Time**
Cognition	Strategic attention: visual selective learning task	(53)	30 min
	Innovation: pictures	Developed at Center for BrainHealth	
	Innovation: high-level interpretation (TOSL)1^*^	(54)	
	Abstraction: proverbs	Developed at Center for BrainHealth	
	Integrated reasoning: high-level summary of text (TOSL)	(54)	
	Memory for detail (TOSL)	(54)	
Well-being	Happiness: Oxford Happiness Questionnaire	OHQ; (55)	15 min
	Depression, Anxiety, Stress Scale	DASS-21; (56)	
	Resilience: Connor-Davidson resilience scale	CD-RISC-25; (57)	
	Life satisfaction: Quality of life scale	QOLS; (58)	
Interaction	Social support: Social Support Survey Index	(59)	8 min
	Compassion	Adapted from (60, 61)	
	Social engagement: Social BrainHealth Scale	(48)	
Daily life	Engagement in Meaningful Activities Survey	EMAS; (62)	20 min
	Sleep: Pittsburgh Sleep Quality Index	PSQI; (63)	
	Metabolic Equivalents: Cardiorespiratory Fitness	CFEQ; (64)	
	Estimate Questionnaire		
	Outlook: BrainHealth Appraisal Questionnaire	Developed at Center for BrainHealth	

* *Test of Strategic Learning*.

The cognitive assessments consist of a battery of measures of complex text, which do not have ceiling effects and are a robust measure of cognitive aging. The cognitive evaluation looks at complex thinking capacities—such as reasoning, innovation, strategy, and memory. The well-being evaluation taps into an individual's emotional sense of self. This includes questions about quality of life, level of happiness, levels of stress and sadness, and emotional resilience. The social interaction evaluation looks at an individual's social vibrancy and quality of relationships, specifically, how they feel about their social support networks and the meaningfulness of their social engagements. The daily life evaluation monitors the complexity (depth and breadth) of daily responsibilities, habits, and challenges. These questions seek to understand how individuals optimize life circumstances and habits.

**Figure 3:**
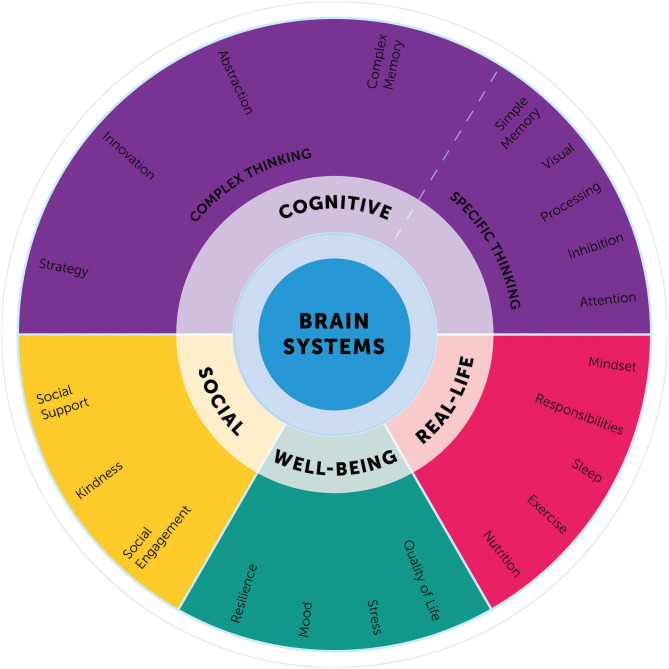
BrainHealth component wheel.

Whereas, many of these individual components of the BrainHealth Index are not novel, what has not been done before is integrating and interpreting these aspects as a single composite index. The BrainHealth Component Wheel illustrates the complexity of these domains (Figure 3) and lays a foundation to build a model illustrating the interrelated, dynamic nature of the components.

The Component Wheel was designed not only to clarify the assessment domains, but also to educate participants regarding the myriad of paths to increase their brain health literacy. The goal is for participants to embrace a perspective that brain health encompasses habits and behaviors working in concert across the above-mentioned domains and that strength building in one domain may generalize to gains in other dimensions. On the downside, participants also learn that losses may follow a similar but reverse pattern with decrements in one domain having a deleterious effect on others.

### Coaching

Three, 30-min individual coaching sessions with a clinician were offered to participants using video conference or telephone calls. The objectives of the coaching sessions were to review the participant's BrainHealth Index, provide awareness to improve literacy regarding the brain's potential to be modified by experience and habits, and to provide direction, guidance, and support related to the online cognitive training, stress solutions, and sleep advice.

### Training Protocol

This was a non-randomized, phase one pilot intervention trial designed to examine the neurocognitive and real-life (emotional well-being, social interaction, and daily function) benefits from an online training protocol. The current offerings were selected based on three decades of scientific investigations, including reported clinical trials showing that strategy training can yield significant gains in the multiple aspects contributing to brain health [e.g., (33, 65)]. These areas of positive change after in-person delivery of *Strategic Memory Advanced Reasoning Training* (SMART, described below) include: cognition (i.e., strategic attention, integrated reasoning, innovation), well-being (reduced depression, stress, anxiety), and real life function (increased quality of life and complexity of life work). These positive behavioral changes were correlated with significant changes in cerebrovascular brain metrics such as brain blood flow, neural connectivity, and neural efficiency (31, 32, 49–51, 66–68).

What was not tested in prior trials was whether the BrainHealth Index and strategy trainings could be delivered through an online platform with similar efficacy and ease of use across the lifespan from teens to late life. The limitations of in-person assessments and trainings include reduced accessibility and high costs—factors that reduce the ability to meet public health demands. A dynamic online platform affords not only increased accessibility to the personalized information and tools provided but also facilitates greater adoptability (i.e., continued engagement) and scalability.

Training consisted of tactical brain strategies that are applicable to a multitude of circumstances, decisions, and goals. Participants completed 9 self-paced training modules over the 12-week period (Table 2). Modules 1-4 were the online, self-paced version of SMART, an evidence-based cognitive training protocol that is strategy-based, rather than content-specific (31, 32, 65). SMART includes tactical brain strategies of Strategic Attention, Integrative Reasoning, and Innovation as a guide for engaging in deeper-level innovative thinking across real-life activities.

**Table 2:** Description of self-paced training modules.

**Module**	**Description**	**Time**
1. SMART 1	Provides strategies and interactive acitvities on how to block irrelevant information and focus on key priorities (strategic attention). Example: Prioritize how you spend your time based on cognitive effort—each day identify top two tasks that requier the most deeper level thinking.	30 min
2. SMART 2	Includes strategies and interactive activities on how to abstract big-picture concepts from information to better inform real life decisions (integrated reasoning). Example: Extract key concepts from incoming information vs. trying to take in and remember everything.	30 min
3. SMART 3	Includes strategies and interactive activities on how to generate multiple and diverse perspectives/interpretations to strengthen mental flexibility (innovation). Example: Identify multiple alternative ideas/perspectives on divisive issues.	30 min
4. SMART 4	Consists of real-life application scenarios where participants can practice dynamic implementation of the strategies from SMART 1-3 (strategic attention, integrated reasoning, and innovation) in a cohesive manner. Example: Think about and prepare for a difficult conversation with someone you care about (considering their perspective, identifying the real issue at hand etc.).	30 min
5. Stress Solution 1	Presents the physiological and neurological response to stress, as well as cognitive strategies (linking with SMART) to manage and reframe stressors. Example: Reframe your perception of your response to a difficult situation from anxiety to excitement.	20 min
6. Stress Solution 2	Includes accessible techniques to help “recharge your battery” in times of stress or fatigue, as well as education on lifestyle factors that can positively impact our overall health. Example: Take several short breaks throughout your day.	20 min
7. Stress Solution 3	Provides research on the benefits of mindfulness, meditation, and healthy sleep habits, as well practical tips on how to practice each one (linking with SMART). Example: Participate in a meditation exercise.	20 min
8. Sleep	Presents research from Dr. Russell Foster on the science behind sleep, the brain impact of poor sleep, and practical tips for improving one's sleep habits.	20 min
9. COVID-19 Information	Safety tips from an emergency medicine physician on how to protect ourserlves and others during the COVID-19 pandemic.	5 min

*Modules 1-7 consisted of videos teaching tactical brain strategies and application exercises. Modules 8-9 were informational videos only*.

Strategic attention strategies help reduce information overload and improve one's ability to block out irrelevant information to better focus and prioritize what really matters. Integrated reasoning strategies target the ability to abstract meanings from specific key details (be it from a situation or information), to interpret them within a broader context of world knowledge, and to create global themes and take away messages that remain relevant to the information or task at hand. Innovation strategies guide people to generate multiple, diverse perspectives and seek multiple solutions to better inform decision-making. Participants are provided exercises to practice real life tasks that incorporate strategic attention, integrated reasoning, and innovation strategies as often as possible within the context of their own daily responsibilities and relationships. The goal is to make this type of thinking habitual by processing information at a focused, calm, and deeper level and to make innovative cognition intentional.

After engaging with the tactical brain strategies delivered via the online SMART modules, participants progressed to training modules 5-7 (Stress Solutions), which linked the SMART principles with stress management research and techniques. Specifically, the Stress Solutions modules expand upon the SMART strategies to manage stress, build resilience, and make healthier lifestyle choices. Participants learn and practice skills in awareness, self-regulation, and emotion regulation through attentional focus exercises grounded in mindfulness meditation. Module 8 focused on information about sleep and how to improve one's sleep hygiene. Module 9 was an informational message from an emergency medicine physician about what steps to take to protect self and others from contracting or spreading COVID-19.

### A BrainHealth Index Prior to Pilot Data Collection

As it was necessary to deliver a quantifiable measure of BrainHealth to our participants at two time points throughout the study, we developed a preliminary Index that would be meaningful to both participants and coaches. By doing so, we could evaluate our online system and real-time coaching and participant feedback without the need to wait until the end of our pilot study. To reiterate, this was an important aspect of the first major goal of the pilot study itself. Therefore, prior to data collection, our preliminary BrainHealth Index was a composite of the measures listed in Table 1, covering the four broad domains of brain health—cognition, well-being, social interaction and daily life. Each measure, *z*_*i*_, was first converted to a common percentile scale *z*_*i*_→*P*_*i*_ and the Index was calculated as $\overset{m}{\underset{i=1}{\sum }}w_{i}P_{i}$, such that $\overset{m}{\underset{i=1}{\sum }}w_{i}=1$. That is, our preliminary BrainHealth Index was a weighted average of transformed sub-domain measures from the BrainHealth component wheel (Figure 2). The transformations were derived as {*P*_*i*_ = 100*p*_*i*_:Φ(*z*_*i*_) = *p*_*i*_}, where Φ(*z*_*i*_) is the cumulative distribution function of *z*_*i*_. Each of the Φ(*z*_*i*_) was determined by one of three methods: (1) Within the cognitive domain the Φ(*z*_*i*_) were estimated empirically, as we have over 1,000 individuals who have taken BrainHealth physicals at the Brain Performance Institute, University of Texas at Dallas; (2) For measures in the other domains, with the exception of the Cardiorespiratory Fitness Estimate Questionnaire [CFEQ; (64)], the Φ(*z*_*i*_) were obtained theoretically using either Gaussian, gamma, or negative binomial distributions whose respective parameters yielded *z*_*i*_ to match distribution statistics given in the references that described or utilized the measure; and (3) Finally, we obtained Φ(*z*_*i*_) for metabolic equivalents, derived in the CFEQ, from a bootstrap distribution obtained using the sufficient statistics of the individual variables that contribute to the model-based calculation of metabolic equivalents.

Because we had no preliminary data prior to the start of the pilot study, the weights, *w*_*i*_, were fixed by clinical consensus of clinicians working on the project. The consensus of three clinicians with over 10 years of experience in BrainHealth Physicals assigned 50% of the weight to the cognitive domain, as these measures have been studied extensively at the Center for BrainHealth, University of Texas at Dallas, showing associations of functional brain changes with training utilization and the measures in the cognitive domain. Daily life and social domains were also assigned relatively higher weight, as these domains represent long term function. However, as noted above, one important goal of our Phase 2 and Phase 3 studies that will follow this pilot is to obtain data-driven calculations of brain health indices. As the raw data emerge from the factors mentioned above, from additional measures of cognition (e.g., processing speed, working memory, sustained attention, inhibition) and from other experimental measures being collected, we will be afforded the opportunity to discover those particular measures that provide the best contribution to, and assessment of, brain health. Importantly, these discoveries will be grounded in a neurobiological and biophysical basis, as a large percentage of participants will also have measures from wearables and from functional and structural brain imaging. Machine learning will guide the mapping of these physical measures onto the space of our online assessments, which will serve both to calculate relevant indices of brain health and to justify the calculation based on physiology.

### Development of a BrainHealth Index From the Pilot Data

Our preliminary BrainHealth Index served an important function for our pilot study. It enabled our participants to receive immediate feedback from their assessments, and it enabled our coaches to offer personalized coaching sessions to participants in real time. Both of these were essential for an engaging user experience and the development of a successful online platform with which we can proceed to Phases 2 and 3 of the BrainHealth Project.

To address our second major goal of Phase 1 of the BrainHealth Project, the data collected from this pilot study was used to develop a modified BrainHealth Index, one that does not rely on fixed *w*_*i*_ by consensus but, rather, one that allows estimated *w*_*i*_ for variables on a natural scale. This was accomplished by fitting an exploratory factor model of the inter- and intra-domain correlations of online assessments. Specifically, we were interested in the relationships of 3-month change measures for each assessment since we posited that measures adaptable to change following training and coaching are those most relevant to plasticity changes in the brain over time. Therefore, a latent factor model of the correlations among change measures was estimated and utilized to obtain appropriate variable weights to transform individual assessments to factors.

## Results

### Demographics

Our initial pilot sample contained 180 participants who completed their online baseline Assessments; 174 were assigned the set of training modules through the dashboard portal. The completion rates of training modules are recorded (described in more detail below), in brief, 144 participants completed their 3-month follow-up assessments, whether or not they completed the training modules. Our retention rate was 80% (see Figure 1).

Table 3 lists some of the demographic characteristics of our initial sample of 180. The age of participants ranged 18–87 with a median of 55.9 and interquartile range (IQR) 33–78.8. Although our sample contained a large proportion of women (72%) and a highly educated cohort (87% with at least a Bachelor's degree and 41% with post-baccalaureate degrees), the age distributions were similar across gender and higher-education categories. For 13% of our sample without at least a Bachelor's degree, the age distribution was bimodal with a median 21.3 for the sub-cohort who are still in college and a median of 63.5 for those who are not in college.

**Table 3:** Demographic characteristics of our sample (*n* = 180).

		Age1^*^ x Gender	Age1^*^ x Education	**Gender x Education**	
Age1^*^	55.9 (22.9)				
Female	129 (0.72)	57.4 (20.6)			
Male	51 (0.28)	49.6 (31.7)			
				**Female**	**Male**
< Bachelor's	23 (0.13)		31.8 (41.3)	14 (0.61)	9 (0.39)
Bachelor's	83 (0.46)		56.9 (20.3)	58 (0.70)	25 (0.30)
>Bachelor's	74 (0.41)		56.8 (24.1)	57 (0.77)	17 (0.23)

n (proportion) except

* *median (IQR)*.

### Preliminary BrainHealth Index

**Figure 4:**
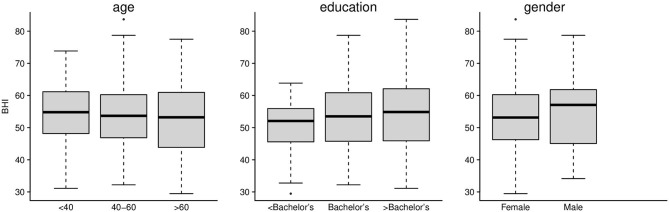
Preliminary BrainHealth Index at baseline for age, education, and gender. The BHI distributions were similar across these demographic categories.

We first assessed whether or not the baseline BrainHealth Index and the change in the BrainHealth Index over 3 months depended on demographic sub-groups, which could indicate potential confounds in our analyses. Baseline characteristics of the preliminary BrainHealth Index by demographics are shown in Figure 4. The median value was 54 overall (IQR 45–61), and the distribution of the baseline Index was similar across age, education, and gender (Figure 4).

**Figure 5:**
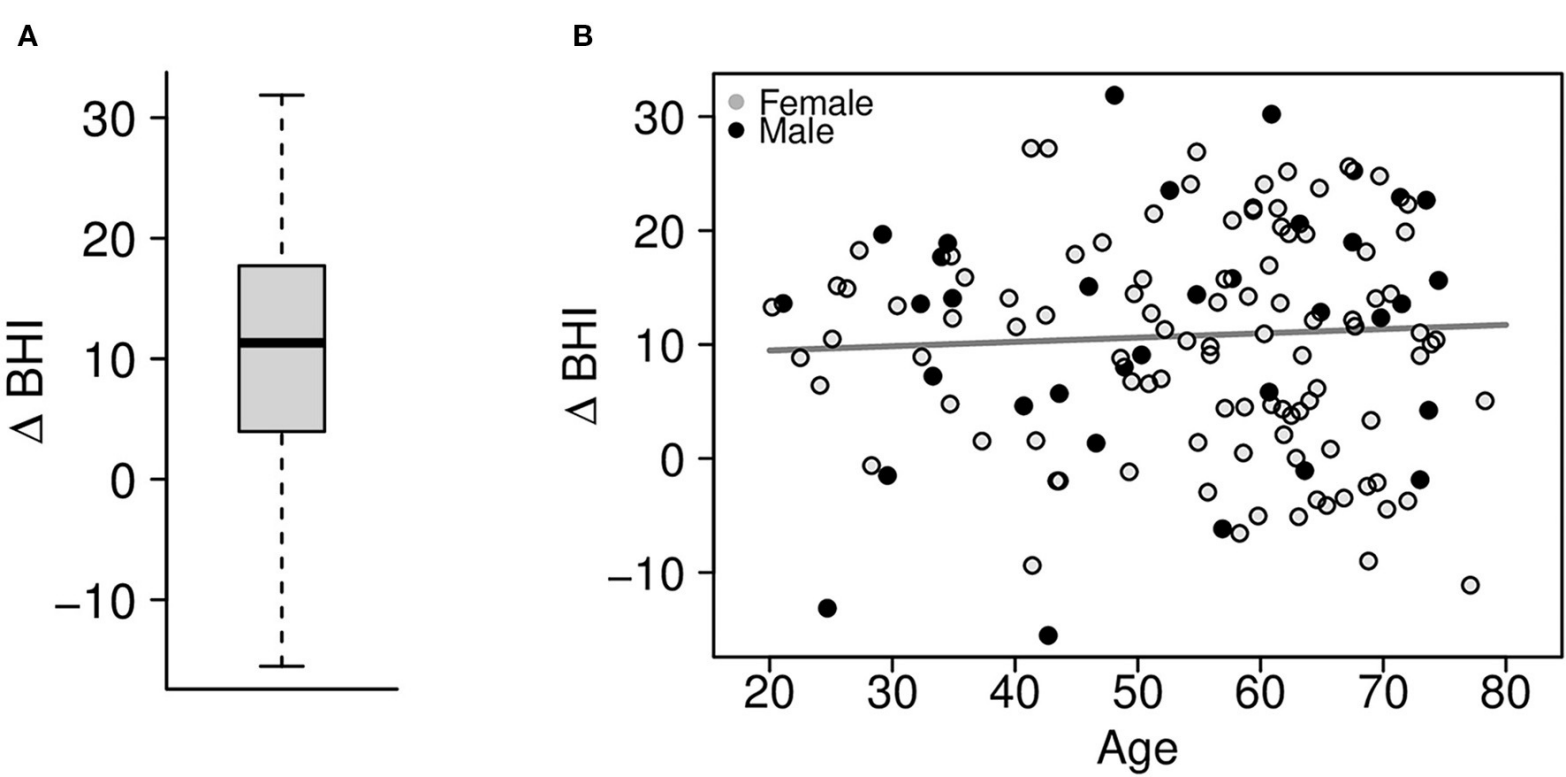
**(A)** Change in the preliminary BrainHealth Index after 3 months with a mean gain of 10.3 units. **(B)** Regression of the change in the BrainHealth Index on age by gender (shown in gray scale) shows that gains do not depend on either of these attributes.

After 3 months following self-paced training and engaged, personalized coaching sessions, the change in the BrainHealth Index (Δ BHI) had an average gain of 10.3 points across the entire sample (Figure 5A, *p* < 0.001, d = 1.03, 95% CI 0.83–1.25). Moreover, 75% of participants showed at least a 5-point gain in the BrainHealth Index over 3 months. As with the baseline BHI values, the change in the BrainHealth Index also did not depend on age (*p* = 0.55), education (*p* = 0.11), or gender (*p* = 0.45). Figure 5B demonstrates the regression of Δ BHI on age. The average change in the BrainHealth Index is near 10 units across the age range and for both male and female participants.

All participants were assigned the training modules (Table 2) as well as informational content about sleep and COVID-19 for a total of 9 assignments, taken sequentially. Of the 80% of participants who completed their second assessments after 3 months, not all of them completed the training modules. We expected that there would be considerable variability in the amount of training participants were willing to complete. Therefore, another important question that we asked was whether the gains in the BrainHealth Index depended on self-paced training utilization, which also served to indirectly assess two potential confounds, namely practice effects and participant motivation.

**Figure 6:**
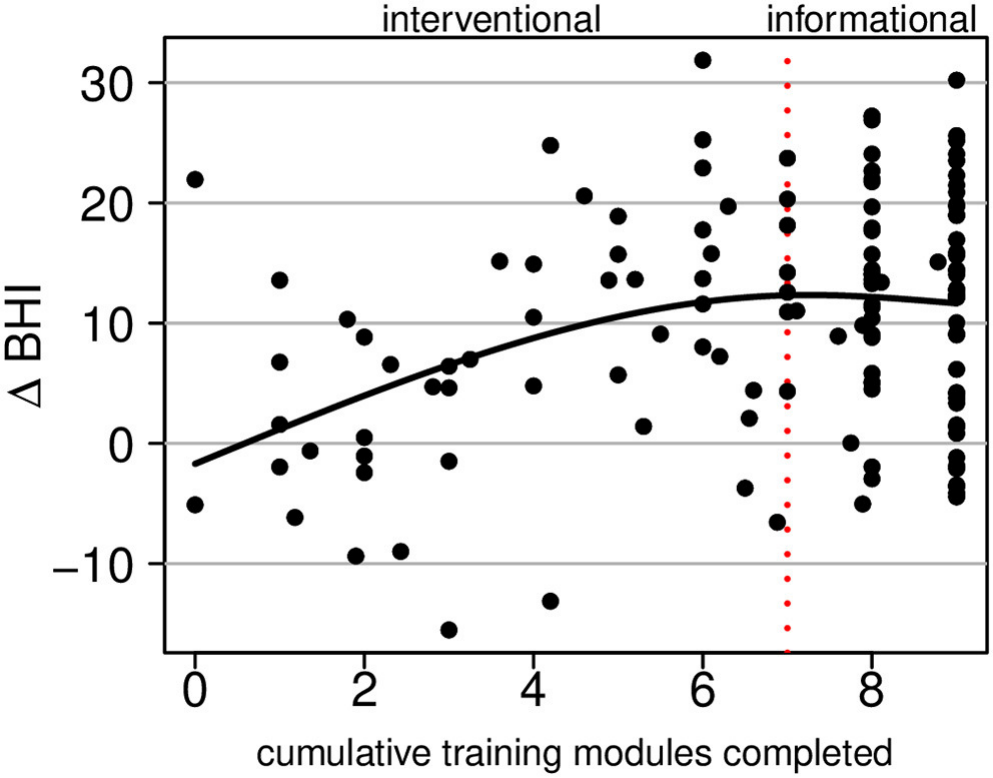
Regression of the change in the BrainHealth Index on the number of cumulative training modules completed by the participant. The first seven modules constitute interventional training; the remaining two are informational only.

Figure 6 shows change in the BrainHealth Index as a function of training utilization, which was estimated by the number of cumulative training modules completed, beginning with the four cognitive training modules and followed by the stress modules, then informational content. There are fractional values for the number of cumulative training modules completed because many participants did not fully complete modules (all activity, including time spent, was recorded).

Superimposed on the scatterplot in Figure 6, we show a conditional average, which was estimated by a regression of the change in the BrainHealth Index on a 3-degree-of-freedom natural cubic spline basis. The curve shows that there is no increase in the BrainHealth Index on average when participants completed none of the training assignments. Conversely, for those that completed the cognitive training modules and nothing further (i.e., 4 modules completed), the average increase in the BrainHealth Index was 8 units (*p* = 0.005, d = 0.98, 95% CI 0.79–1.21). If participants, additionally, completed the stress modules (dashed red line at 7 modules completed), the average increase in the BrainHealth Index improved 4 units further (*p* < 0.001, d = 0.43, 95% CI 0.25–0.61). However, if participants completed the rest of the training assignments beyond the first seven, there was no additional increase in the BrainHealth Index on average, as seen in Figure 6 by the flattening of the regression curve.

The fact that the regression curve in Figure 6 is relatively linear between the values 0 and 6 (training modules completed) suggests that gains in the BrainHealth Index are likely not solely due to practice effects. If gains were solely due to practice effects, one would expect a horizontal line at the overall average change in the BrainHealth Index (10.3 units), regardless of training utilization. Moreover, if the increase in the change Index were due only to participant motivation, independently acting upon the change Index and the training utilization, one would expect an increase over the entire interval of training modules completed. However, this is not the case. There is no additional increase in the BrainHealth Index on average beyond 70% of the training assignments completed. Importantly, this observation cannot be explained by a ceiling on potential change because there exists a much larger range of potential change beyond that which is represented by our sample in Figure 6.

### Components of the BrainHealth Index

**Figure 7:**
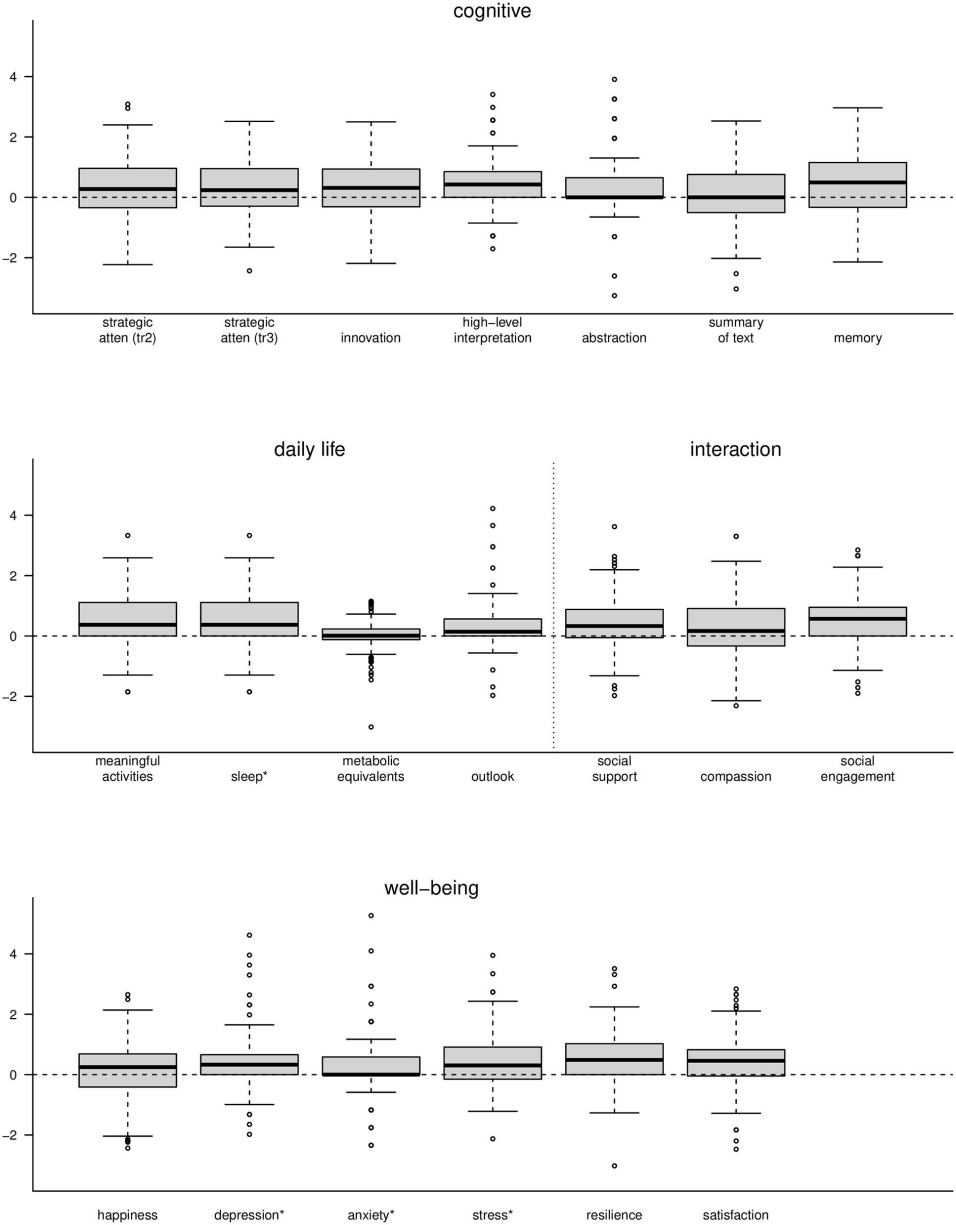
Boxplots of change measures for each of the components of the BrainHealth wheel. Each measure was scaled for presentation on a common axis. *The measures for sleep, depression, anxiety, and stress are shown with opposite sign.

The contribution to the increase in the BHI over 3 months was due not only to measures in the cognitive domain, as one might expect following cognitive training, but also from the rest of the measures in all domains of the BrainHealth wheel. These included those measures within the domains of well-being, daily life and social interaction, even though training and coaching strategies focused largely on the cognitive components. Figure 7 shows box plots of individual measures within each of the BrainHealth wheel domains on scaled axes. Many of these measures demonstrate improvements—some as much as 75% of the participants showing improvements—including the sleep index and the depression, anxiety, and stress indices from the DASS-21. Improvement for these measures is indicated by a decrease from baseline (although we reversed their sign in Figure 7 so that change would be commensurate with the other measures).

These observations are important because not all individuals improve their BrainHealth Index by a similar route. Some showed gains due to increased resilience and decreased anxiety, for example, whereas others improved due to increased cognitive flexibility and social engagement. One of the primary goals of the BrainHealth Project (through Phase 3) is to enable individuals to understand their own capacity to improve brain health, by a route that may be specific to each participant and encouraged by tailored coaching. Indeed the mechanism by which an individual achieves improved brain health is multifaceted, and we intend to leverage that variability as we utilize our pilot data and, subsequently, the imaging data we will collect in Phase 2 of the BrainHealth Project to develop individual prediction models and build BrainHealth indices based on biomarker validation.

### A Factor Analytic Model: Toward an Improved BrainHealth Index

**Figure 8:**
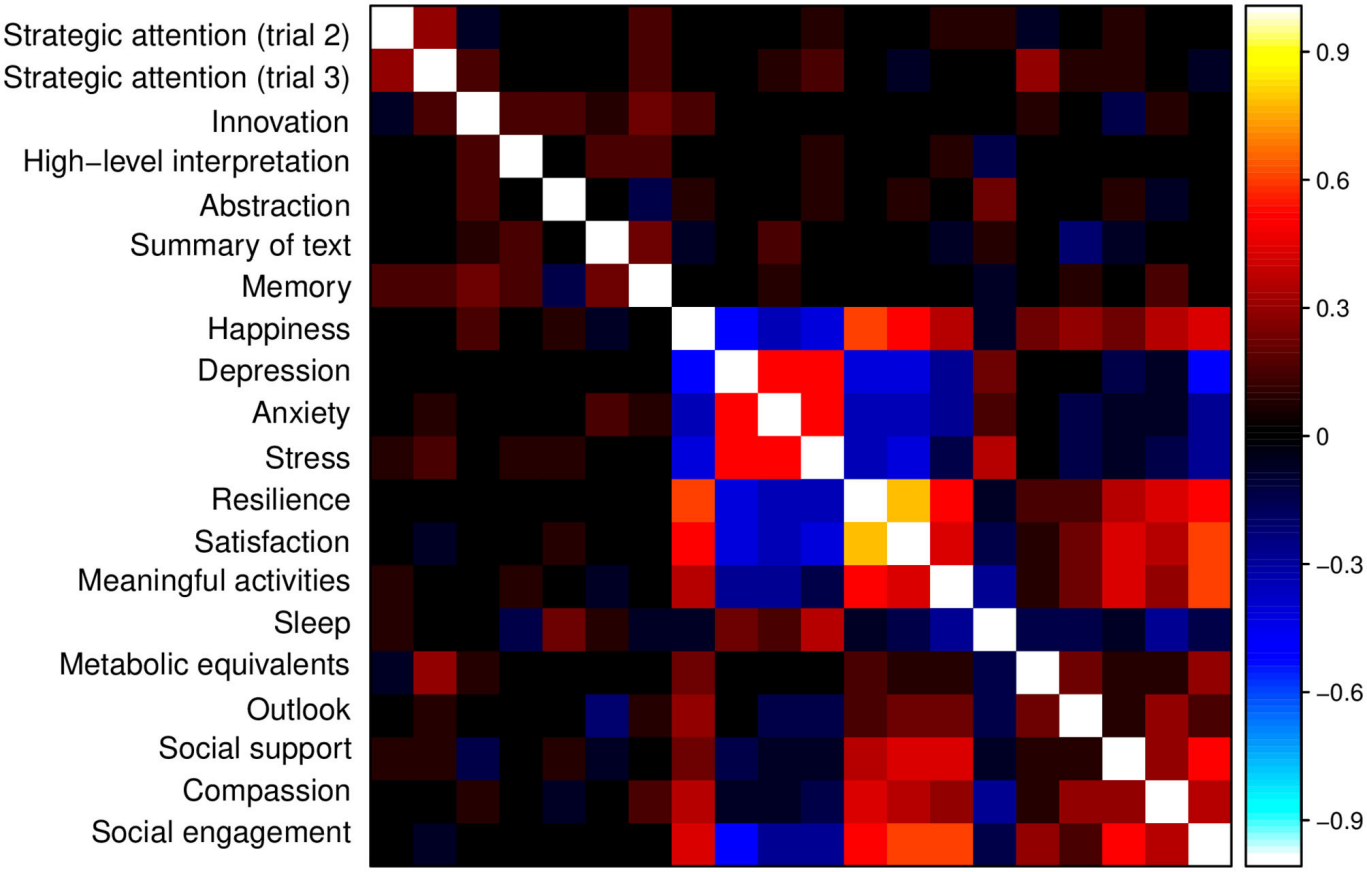
Correlation matrix for the measures from the BrainHealth wheel domains, obtained by online assessments. Across-domain correlations are relatively strong and contribute to latent factors of brain health that incorporate these extended relationships.

The correlation matrix of change measures, shown in Figure 8, suggests that a data-driven BrainHealth Index can be built upon a factor structure from the relationships among the wheel-domain components.

Although many of the stronger correlations are within-domain, as expected, there are also strong across-domain correlations (e.g., across cognitive and social interaction domains; across well-being and daily life domains) that may be important to latent constructs of brain health. These constructs allow for a natural reduction in components and a model-derived set of weights for each component.

**Figure 9:**
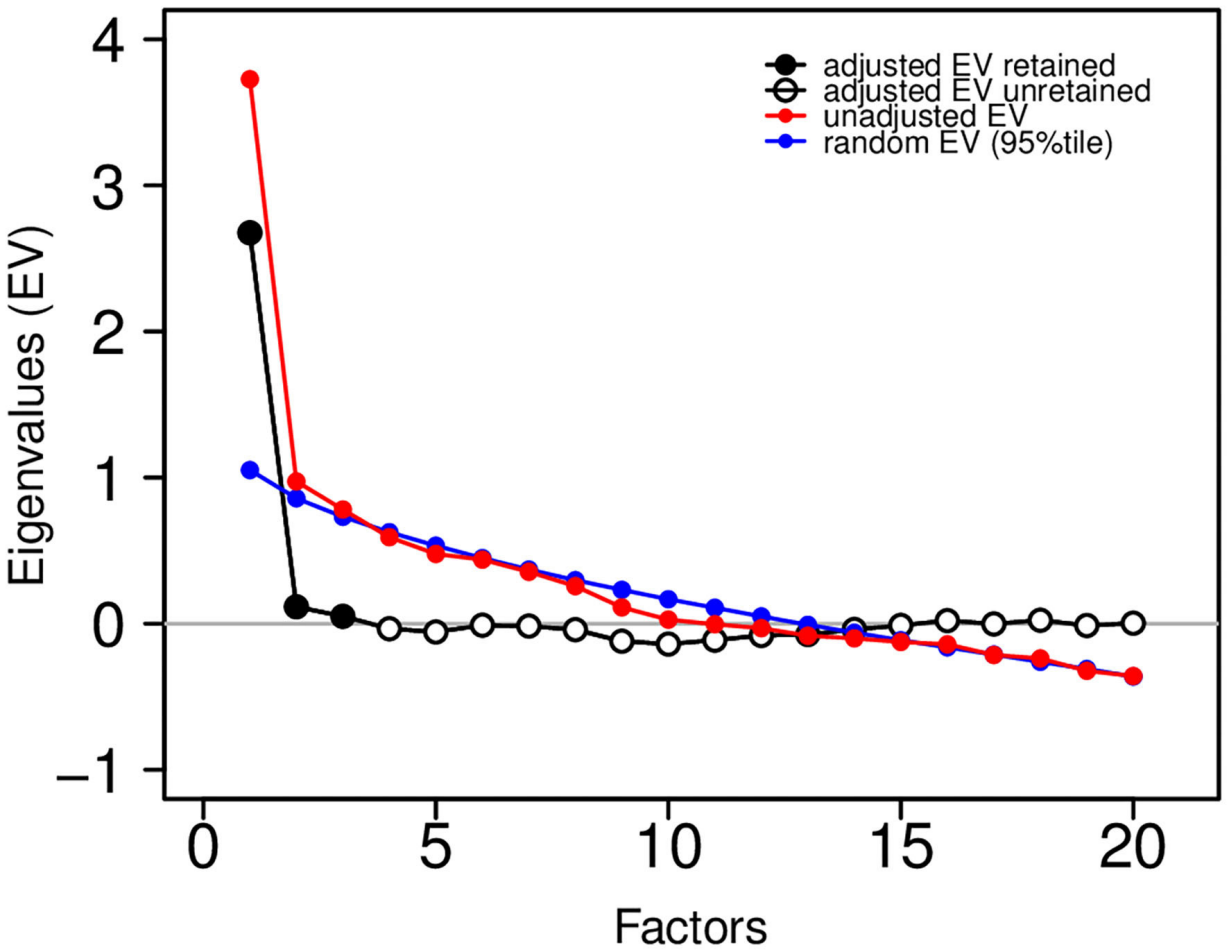
Eigenvalue plot showing unadjusted eigenvalues (in red) and adjusted eigenvalues (in black and white). The factor solution was based on three retained (three adjusted eigenvalues greater than zero). Adjustments were based on the 95th percentile of a random eigenvalue distribution (in blue).

A factor solution for the correlation matrix in Figure 8 was estimated by maximum likelihood using the R statistical computing language (http://r-project.org). We used varimax rotation to find simple structure, if one existed, and we utilized Horn's parallel analysis to estimate the appropriate number of factors for a solution. Figure 9 shows the eigenvalues (EV) from the factor analysis, along with the 95th percentile of the eigenvalue distribution from random factor structure.

The difference between these are the “adjusted” eigenvalues. Those adjusted eigenvalues greater than zero are retained (adjusted EV retained in black; adjusted EV unretained in white). This analysis suggests that a 3-factor solution is sufficient. To assess the adequacy of the fit of the factor solution to the observed correlation matrix, we show the following common fit statistics: RMS of residuals − 0.058; Tucker Lewis Index (TLI) − 0.912; RMSEA Index − 0.036 (95% CI [0,0.061]); Fit (off-diagonal) − 0.912.

The likelihood Chi square was 158.14 on 133 df (*p* = 0.067). RMS of residuals and RMSEA index both <0.08, *p* > 0.05 from likelihood Chi square, TLI and fit statistics both > 0.90 were all taken together as an indication of good fit of the factor solution to the observed correlation matrix as suggested in the factor analysis literature.

The BrainHealth wheel components that contribute to each of the three factors are shown in Table 4. The factor loadings have been normalized and those measures whose loadings are at least 0.200 in absolute value are highlighted in Table 4 to identify the BrainHealth wheel measures that contribute most to each factor. The cut-off of 0.200 leads to the largest loadings, which comprise at least 80% of the normed length for each factor.

**Table 4:** Normalized factor loadings.

**Measure**	**F1**	**F2**	**F3**
Strategic attention (trial 2)	0.046	−0.100	0.139
Strategic attention (trial 3)	0.009	−0.128	**0.322**
Innovation	−0.036	0.017	**0.344**
High-level interpretation	0.037	−0.086	**0.209**
Abstraction	0.083	−0.061	−0.176
Summary of text	−0.060	−0.007	0.178
Memory	−0.044	−0.043	**0.605**
Happiness	**0.240**	**0.245**	0.186
Depression	−0.086	**−0.463**	0.036
Anxiety	−0.021	**−0.506**	0.099
Stress	0.024	**−0.568**	−0.040
Resilience	**0.368**	0.156	0.119
Satisfaction	**0.392**	0.157	0.030
Meaningful activities	**0.385**	−0.036	−0.017
Sleep	−0.054	−0.151	**−0.200**
Metabolic equivalents	0.166	−0.102	0.149
Outlook	0.134	0.017	**0.205**
Social support	**0.390**	−0.145	−0.103
Compassion	**0.274**	−0.057	**0.317**
Social engagement	**0.451**	0.009	−0.094
Cumulative variance	0.144	0.229	0.275
Proportion explained	0.524	0.309	0.167

*Highlighted are those contributing most to each factor*.

Table 4 reveals a near simple structure for the factor solution: Only the measures of happiness and compassion load on more than one factor by the criteria described above, but the simple structure is not the wheel domain structure themselves. Factor 1 is dominated by the measures in the social interaction BrainHealth-wheel domain and those measures in the well-being domain that constitute mental fortitude (e.g., resilience and satisfaction). Factor 2 is derived almost exclusively from mood assessments in the well-being domain—assessments of depression, anxiety, stress, and happiness constituting factor 2. Finally, factor 3 is comprised of cognitive components that measure strategy, innovation, and memory; but contributions from sleep and outlook within the daily life domain and, especially, compassion within the social interaction domain are equally as important to factor 3. The structure implied by this factor solution, then, suggests that a BrainHealth index can be derived from three latent constructs, which we will utilize as testable constructs. This factor structure is the first step to derive a data-driven BrainHealth Index. From this latent structure, we can now calculate a new Index based on combining construct scores for Phase 2 of the BrainHealth Project.

### Participant Responses and Case Illustrations

In this section, we provide examples of participant feedback and two case studies exemplifying the personalized nature of the BrainHealth Index and training response. Table 5 provides a sample of participant quotes regarding the feasibility and ease of use of the online platform. Also displayed are sample participant responses regarding whether the training and information provided impacted their understanding of brain health and their sense of agency related to their brain health.

**Table 5:** Participant responses on feasibility and utility survey.

Ease of use	Everything taught was well-grounded and presented in a concise, easy manner. If someone is serious about improving their brain health, they can easily pick up on one or more of the cues and pursue it more in-depth elsewhere.
	Everyone will learn some things that will improve the quality of their lives. And it's so easy to do.
	I believe that the content was helpful, information, and laid out in a clear and concise way. I was able to easily understand the strategies and apply the ones I believed to be beneficial to my life.
	There are concrete steps that can be taken to measurably improve brain health.
	I like the conceptual framework that seems to suggest that brain health can be developed and nurtured through skills-based training and practice.
Brain health literacy	This is not about playing games to train the brain this is about putting in place lifestyle changes that put you in a position to succeed
	I learned the importance of balancing all of the areas of brain health due to their interdependence on each other. That was very eye opening.
	The relationship between cognitive capacities performance on the well-being and daily life activities. The importance of not ignoring those areas.
	How important good sleeping habits are to my overall brain health.
	Having new/different perspectives is an important part of cognition and brain health as much as things like memory
Agency in brain health	I learned so much about how I can make or break my brain health. I learned specific strategies that I can implement in my everyday life that help my brain stay healthy.
	Overall, I feel a little more relaxed, confident and happier. In addition to sharpening my focus and broadening my perspective, the Brain Health project provided me with some tools that enhance my well-being.
	I can keep improving my brain health no matter how old I am.
	The SMART strategies I learned about and applied during my experience greatly improved my cognitive abilities.
	Individuals who participate have an opportunity to improve their lives through better understanding of how their brain functions and how it can possibly be improved.

**Figure 10:**
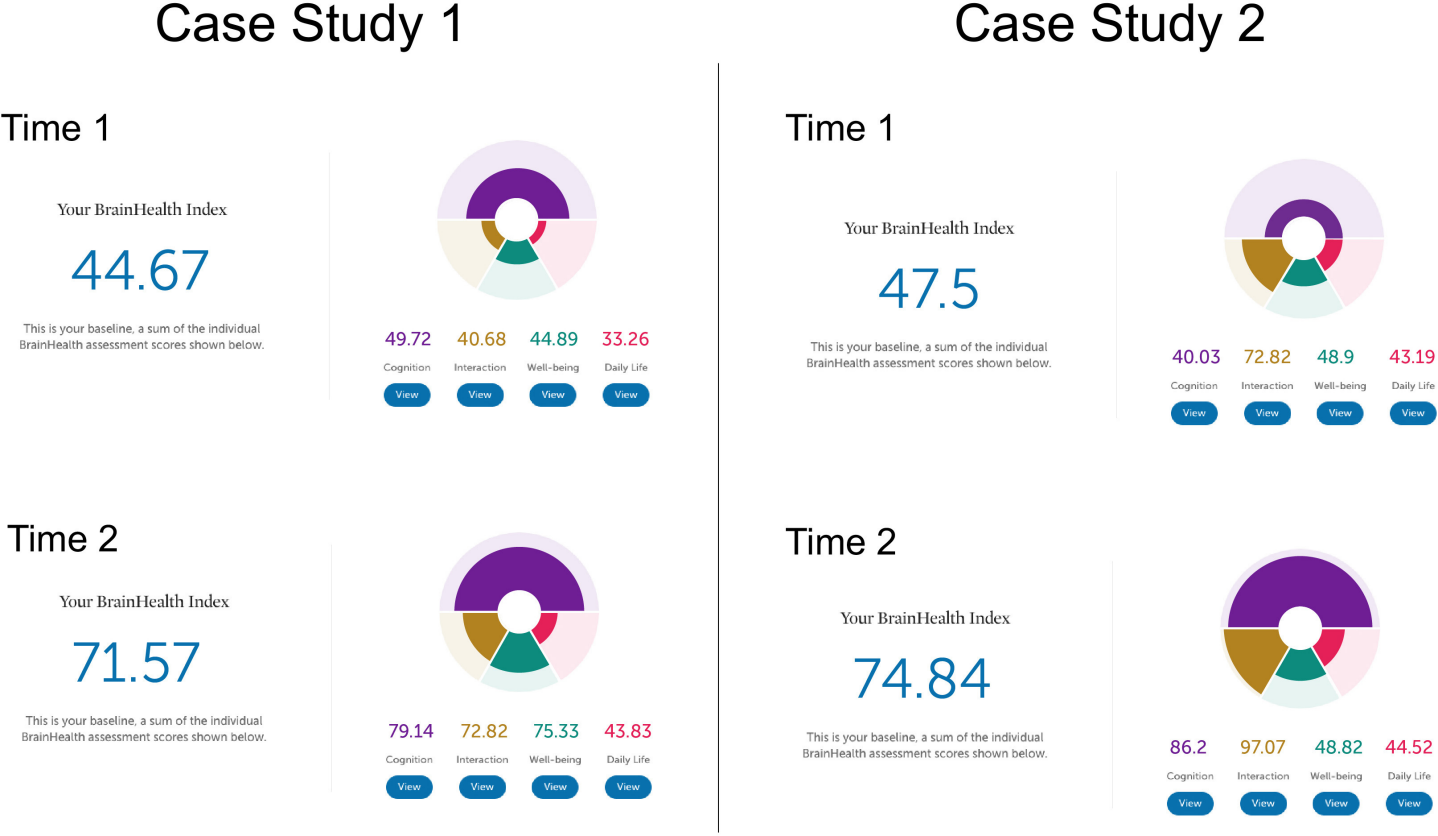
Two case studies.

The following two case studies illustrate how the BrainHealth Index was represented for the user with interpretation provided by the coach to achieve clarity in understanding their results. The case studies show how pre- to post-training performance may be reflected in overall change in BrainHealth Index scores, and how similar change in pre/post scores may be due to changes in different contributing brain health components. Additionally, these case studies are presented to illustrate the opportunities machine learning creates to refine data gleaned from the BrainHealth Index and increase the individual precision and predictions of the various trainings and recommendations offered based on the BrainHealth Index outcomes, especially possible as more data is collected. Refer to Figure 10 for an illustration of the BHI scores and changes from Time 1 to Time 2 for both case studies.

Case study one is a 55-year-old female with a bachelor's degree. She is currently a homemaker. She enrolled in the BrainHealth Project because of her interest in improving her own as well as her family members' brain health and performance. At Time 1, her performance on the Cognitive component of the BrainHealth Index (score of 49.72) reflected difficulty with strategic thinking and fluency of novel ideas. On the Daily Life (score of 33.26), Social Interaction (score of 40.68), and Well-being (score of 44.89) components of the BrainHealth Index (overall score = 44.67), her responses showed reduced purpose, difficulty with sleep, feelings of isolation, and missing her social network despite having good friends and family, and some elevated stress, all due to pandemic circumstances. She reported feeling overwhelmed and having trouble seeing possibilities that existed within the COVID-19 pandemic environment. Her BHI scores mirrored her subjective experience. At Time 2, her BrainHealth Index scores reflected the application of her learnings: in the Cognitive component (score of 79.14), her strategic blocking of unnecessary information improved, as did her fluency for novel, meaningful ideas; in the Daily Life component (score of 43.83), she felt greater purpose and her sleep improved; in the Social Interaction component (score of 72.82), she felt more satisfied with her social networks; and in the Well-being component (score of 75.33), her resilience improved as did symptoms of stress. She stated that she had developed a more strategic approach to tasks in all of the BrainHealth components (overall score = 71.57). She started to prioritize regular contact with family members using video calls and spending uninterrupted time each day with her husband. She also learned how to better process and control her reactions to difficult situations. The overall change from Time 1 (44.67) to Time 2 (71.57) was a 26.9 point gain.

Case study two is a 20-year-old male, with a bachelor's degree, who is working as a full-time teacher. He enrolled in the BrainHealth Project because of his desire to improve his critical thinking skills and to help his students do the same. At Time 1, his Cognitive component score (40.03) showed some reduced abstract thinking despite having good ideas and some reduced memory function. He reported dissatisfaction with his thinned social network but overall satisfaction with his life considering the pandemic environment. At Time 2, his Social Interaction component score increased (from 72.82 to 97.07), as did his Cognitive component score (86.2) related to abstraction, synthesis, and memory performance. He found his BHI scores to accurately represent his subjective report or increased confidence in his abilities. He was engaging in abstract thinking more routinely, and applying those concepts to his life. He had moved in with his partner and felt more socially supported. The overall change in his BHI score was a gain in 27.3 points (from 47.5 to 74.84). We chose to present these two case studies as an example of how similar point value changes can be due to gains in different aspects of brain health, but also to reinforce the value of a holistic measure. Individuals may advance their brain health over the lifetime building different competencies to thrive in the current life context.

## Discussion

This Phase 1 pilot study had two main objectives. The primary goal was to test the efficacy of an online delivery platform across the adult life span (18> years of age), which entailed three sub-aims. The first sub-aim sought to determine use of a remote/Telehealth-Delivery platform to administer the compendium of tests that comprise the BrainHealth Index. The second sub-aim was to assess how feasible and usable a set of training modules would be when delivered through the same remote platform as the BrainHealth Index, as measured by dose response (i.e., percentage of training sessions utilized). We evaluated the utility of online offerings in this Phase 1 pilot trial to complement our previous decades of research that identified positive gains when the majority of components comprising the BrainHealth Index and the trainings were delivered in person. For the third sub-aim, we wanted to evaluate how effective brief “live” virtual coaching sessions would be in guiding participants how to interpret the novel outcomes from the BrainHealth Index, and make progress toward individualized brain health goals. The second main objective of this pilot study was to develop a data-driven BrainHealth Index from an exploratory factor model of BrainHealth constructs that could be tested and validated in the next phase of our BrainHealth Project. Our ultimate goal was to lay the groundwork for a subsequent large-scale effort to establish an objective, repeatable, and interpretable framework to evaluate how the components combine and co-vary to contribute to holistic gains or losses in brain health metrics, at both group and individual levels.

### Efficacy of Online Platform Delivery

The results provide evidence that an integrated measure of brain health, reflected by a BrainHealth Index, can be meaningfully delivered online to individuals across the adult lifespan. The preliminary results and feedback from the participants suggest the composite has the potential to measure changes (whether gains or losses) as well as to chart performance stability over time with or without intervention protocols. As discussed in Methods, the weights used in the preliminary BrainHealth Index for this pilot effort was derived from clinical judgment/experience with a combination of previously validated and standardized measures of cognitive, emotional, social and everyday life function. The provision of a single index, comprised of four components, may have been a factor in motivating participants to engage in this entirely remotely-administered program. We suggest that individuals have different curiosities that may be self-motivating when they have multiple routes to improve global level of brain performance. These different metrics of human brain function typically have not been measured at all and if they were, the domains were assessed by different experts and never integrated or interpreted as to their covariance despite the fact that a strong interdependence exists. The combination of a multitude of measures into a single index in this effort is entirely novel.

Typically, efficacy of intervention protocols, including pharmacological and neurobehavioral treatments, are assessed primarily with cognitive measures, whereas those aimed at emotional problems, such as mood or anxiety, do not typically measure cognitive function. Yet, many studies have shown that emotional, cognitive, social, and everyday behaviors mutually interact and influence each other to such a degree that the combination of components into a single index is entirely appropriate and perhaps more instructive. Anxiety, for example, degrades attentional capacity (14), and low mood and depression also worsen cognitive function, including attention, and executive and memory processes (17). Poor cognitive abilities, particularly low attentional control, on the other hand, lead to emotional regulation problems (20). People tend to experience problems in life in a holistic way, not one compartmentalized into somewhat arbitrary domains such as emotion and cognition. This means that having a single derived index representing this overall state may not only be meaningful, but also potentially motivating, as was implicated by the 80% adherence rates during this study. Real-life examples include an individual who, by learning to reframe her stressors, improved her sleep and cognitive flexibility, and another individual who improved her social interactions, stress, and resilience by utilizing cognitive tools to increase innovative capacity and become a stronger “possibility thinker.”

Simple measures related to heart health such as blood pressure or blood cholesterol are widely understood in peoples' minds and as such serve as motivating targets for improvement through lifestyle changes such as exercise, nutrition, and stress. As we make progress in evaluating the BrainHealth Index in larger samples over longer periods, we may find that individual or combined parameters from the Index may be predictive and targeted as preventive measures, at either a group or individual level. The creation of a single BrainHealth Index offers a systematic way to start exploring such possibilities in the domain of brain health. The provisional Index developed in this study met the objective of being deliverable, repeatable, and acceptable. Its promising scalability with an online delivery platform is of particular importance to meet the first aim of the study, namely whether we can address brain health status and overcome in-person challenges of participation that were brought to the forefront during the COVID-19 pandemic.

### Usage and Acceptability of Training Modules

The training offered in this program was also unique because, unlike other methods, it focused entirely on training tactical brain strategies (e.g., integrated reasoning, innovation) that can be directly adopted in real-life activities rather than training of isolated domains of specific cognition processes (e.g., attention, working memory). Similarly, the stress modules of the program used low-demand, micro-learning video instruction of tactical strategies with a highly positive tone unlike more clinically-oriented stress programs. The cognitive training was entirely focused on “top-down” strategic approaches to thinking, i.e., innovation, big-idea synthesis and strategic memory. We acknowledge the potential advantage of incorporating additional “bottom-up” training programs aimed at enhancing functions such as speed of processing (41, 69). Promising data from these latter approaches suggest that the effectiveness of the current program could be further enhanced by adding these to the menu of modules. We will address this additive value as our participants continue to be followed and these modules are added.

A majority of participants, regardless of age, gender, or education, showed significant gains in the BrainHealth Index based on training usage. Specifically, the results showed a significant training dose-response relationship on BrainHealth Index gains. The more training modules individuals completed, the higher were their gains on the BrainHealth Index, at least for the tactical training modules vs. the purely informational modules. The fact that there was no such dose-response relationship for the purely informational modules completed offers a partial quasi-experimental control which we cautiously interpret as a real training effect on the BrainHealth Index. Pre-existing motivation could have been a confounding variable explaining both the BrainHealth Index increase and the high program engagement. However, had that been the case, then engagement with the informational modules should have continued the rise in the Index gains—but the gain curve flattened for these, so motivation is unlikely to be a factor.

One might argue that the gain could be due to practice effects. Practice effects can be cautiously ruled out as a second potential confound because of the dose-response relationship between active training-module utilization and brain health gain. Had improvements in BrainHealth Index been entirely practice-related, then they would have increased independently of module usage.

### Live Virtual Coaching

No previous endeavor has combined online cognitive, emotional, and lifestyle interventions with real-person, virtual coaching. The ability of the coaches to incentivize participation by feeding back strengths and weaknesses in the components of the BrainHealth Index is an additional factor that may help explain the high compliance rate of 80%.

The role of coaching in these possible training-mediated brain health improvements cannot be underestimated. Combined with a simple, incentivizing metric to work toward, and training modules with very high face validity and real-life relevance, the coaches were able to motivate and direct participants to call upon areas of relative strength to shore up and strengthen areas of weakness with only two 30-min sessions. The first session served to help ensure the participants make sense of the personalized BrainHealth Index and set goals. The second was for the coach and participant to work together to find ways to apply the tactical brain strategies to their individualized life context. The quality/experience of the coaches will be a major factor to address as the number of coaches increase nationally and, eventually, internationally. We have developed a coach training manual/process that is being tested to determine the level of expertise necessary for a coach to be effective, recognizing that quality will vary across coaches just as it exists for physicians. We will also create different exercises and train coaches that are appropriate to different cultural groups.

We propose that these pilot data and participant responses support the simplicity and clarity of the BrainHealth Index. Furthermore, the accessibility and acceptability of the online training modules across the adult age span suggests that the relatively low-cost of training the large cohort of coaches needed to scale this program, for the millions of people who would benefit from it, is entirely possible. We estimate that one coach can work with ~500 participants a year, which is a small expense compared to the cost savings in keeping a person's brain functioning at fuller capacity longer. The major hurdle will be training, monitoring, and managing the large numbers of coaches needed to scale the BrainHealth Project.

### Application During a Pandemic to Strengthen Brain Health

The timing of this pilot trial came at an opportune moment, given it coincided with the outbreak of the COVID-19 pandemic, necessitating remote participation. Smith et al. (5) argue that the COVID pandemic is causing global misery with social isolation, loss of jobs, and livelihoods, unpredictable forces exacerbating stress, depression, and anxiety. Indeed, the majority of our participants reported notable deleterious impacts from the pandemic ramifications, even though few had contracted the virus. Smith and the international team of authors claim that the time is urgent to catalyze proactive efforts to invest and focus attention on brain health to help strengthen brain health, intercept early concerns and vulnerabilities, and develop measurable strategies for brain skills to rebound from losses given COVID has created a brain health crisis. We are beginning to realize that resilience from massive disruptions can occur when brain health is foregrounded as a key driver and solution.

These pilot data offer promise that steps can be taken to reinforce brain skills even under times of unprecedented stress and unpredictability. The fact that 75% of the participants from ages 18 to 80 showed at least a 5-point increase, with a mean gain of 10 points in their BrainHealth Index, during one of the worst times in our global economy and public health crisis offers hope. This upward potential on a quantifiable BrainHealth Index composite suggests that individuals may be able to build resilience and perhaps even intercept losses before they become clinically full-blown. Intercepting losses will require longitudinal follow-up, which is already underway. This pattern of comparable baseline and gains across the lifespan challenges the long-standing perspective that peak brain years are in the 30s and that neuroplasticity weakens with age. Clearly, certain aspects of cognition, such as speed of processing and working memory, decline with age (70, 71). However, we suggest that a holistic approach to brain health will be fruitful. This approach takes into account the vast reservoir of brain capacities (such as social adeptness, innovative thinking, and life purpose to mention a few) and provides individuals guidelines to engage multiple, not singular, pathways to strengthen brain health to thrive in life. A siloed/segmented approach to brain health fails to take into account the complexity and vastness of capacities to adapt and continue to thrive.

It is often times of crisis that spark an openness to new approaches that improve life. Because of the isolation brought about by the pandemic, people have become much more open to e-health platforms. If we had tried this online aspect of the BrainHealth Project just 1 year ago, it may have met with resistance from different groups. In issues of the brain, the online offerings may provide a more anonymized way to seek help rather than ways that reinforce the stigma of identifying deficits and labeling something as wrong, instead empowering participants to be proactive about building their brain capacity.

### Validation Using Machine Learning

With the exploratory latent constructs of BrainHealth developed in this pilot study, which is part of Phase 1 of the BrainHealth Project, we will now proceed to the second phase of the Project to incorporate brain-imaging metrics in an independent sample of 200 participants. All aspects of Phase 2 will mimic Phase 1, including training modules and personalized coaching sessions, but the BrainHealth Index will be calculated using the three latent structures developed in this pilot. Most importantly, we will add imaging metrics using MRI such as regional brain volume, regional blood flow, functional connectivity, and structural connectivity. Brain imaging metrics will be collected at baseline and 6 months post-training, in addition to the online assessments from Phase 1. The purpose of adding the imaging metrics is to validate the BrainHealth constructs (and hence the Index itself) by finding the set of metrics that can best predict the construct domains.

Although much is known about associations between various imaging metrics and clinical measures, these are predominantly in the context of pathology (e.g., clinical depression, dementia). Far less is known about these associations for healthy individuals with a goal of maintaining or improving their brain health, similar to the goal of maintaining or improving physical fitness as we age. This is a Big Data problem. With the success of machine learning in many different fields of study, including imaging-based prediction, we plan to leverage learning algorithms to help us find those relevant biomarkers that can best predict our testable constructs of BrainHealth.

Our first plan of attack is to develop locally connected deep learning networks that will take in imaging metrics across different modalities as features and propagate those through several layers, at the end of which is an output that represents one of the BrainHealth constructs. There will be a network for each of the BrainHealth constructs, and the algorithm will learn the imaging features that predict, as closely as possible, the measured construct. How the final Index is weighted from the construct domains will depend on how robust each set of learned features predicts the network outputs. More weight is given to the more efficient network-based prediction, as there would be more evidence of a physiological basis for the construct itself in that situation.

The strategy briefly described above will serve two important goals of the BrainHealth Project. First, to fulfill the goal of Phase 2, it will validate the BrainHealth Index as a quantifiable measure of a state of an individual's brain health that can be reliably tracked over time. Secondly, successful prediction models of BrainHealth constructs will allow a tailored approach to improving brain health for individuals. Just as physical health for different individuals can be improved by tailored means (i.e., precision health), the same would be true for brain health. Coaching strategies could be tailored to individuals for maximal benefit.

### Limitations

The BrainHealth Index is a dynamically-adapting tool for reflecting to an individual their level of functioning and motivating them to make changes to improve it. The BrainHealth Index will also change over time as machine-learning analysis of accumulating multi-dimensional data refines it. This unique strength of the BrainHealth Index also limits its use as a conventional static biomarker, at least during the first period of its iterative development. We regard the advantages of our approach to the BrainHealth Index to outweigh this limitation, however.

The trainings offered in the BrainHealth Project represent a relatively small proportion of the interventions that could and will be offered on this unique, coaching-facilitated platform. For example, the cognitive training consists of “top-down” training focused on high-level executive and attentional abilities and does not yet include some of the robust and effective “bottom-up” training of the type offered, for example by BrainHQ (41, 69). Similarly, our advice on important functions such as sleep, exercise and diet, could be complemented as we integrate reliable data from wearable devices and provide targeted behavior-change focused programs in these domains.

The acceptability of, and adherence to, this training and assessment program to a wide range of socio-economic and cultural groups is not yet established. This serious limitation of the present pilot study is one we propose to address with alacrity.

Finally, this is an “open-label” trial and not a randomized controlled trial. We must therefore be cautious in ascribing cause-and-effect relationships between the training and the observed improvements in the BrainHealth Index. Whereas, this is a significant limitation, the results offer promise as supported by the compelling dose-response relationships between training engagement and BrainHealth Index improvement. Perhaps, this type of clinical trial may offer promise in the future to interventions that show no harm but have been shown in smaller, carefully controlled, randomized trials to be effective. Even more important is to document the persistence of the gains over time. One fact of neuroplasticity is that the brain never stays the same; just as gains are possible, so are losses. Therefore, keeping the brain fit will require continued effort on behalf of individuals. What is promising is that 95% of the people in this pilot signed up for the 10-year study to continue to measure, monitor, and take advantage of trainings to maintain or improve their brain health.

### Future Studies

The current Phase 1 pilot study lays a foundation for the next critical Phase 2 of the BrainHealth Project, which will include the addition of functional and structural imaging measures. In this second phase, we will incorporate machine learning models to map the imaging metrics onto our exploratory factor model space as described above. The purpose of this phase will be to refine and validate the factor structure and to obtain a data-driven BrainHealth Index measure that relates to brain biomarkers.

These two trials, Phase 1 and 2, will inform our scientific efforts as we prepare to launch Phase 3 of the BrainHealth Project with the goal of reaching hundreds of thousands of participants across demographic domains. This larger effort requires multi-institutional collaborations to show reliability and validity of measures as larger numbers of participants are followed over 10 years with semi-annual BrainHealth Index metrics, semi-annual physiologic measurements from wearable data on 20% of the participants, and semi-annual imaging metrics on 10% of the participants.

Future efforts need to greatly extend the demographic, socio-economic, and cultural reach of the populations addressed, to test whether high levels of acceptability are maintained, and if not, how these can be achieved. Finally, future studies should incorporate new evidence-based interventions and incorporate them into the platform. Such an approach is clearly antithetical to a classic randomized-controlled trial where the “treatment” must be fixed before the trial begins and not change as new validated interventions appear. The limitations of current practice randomized trial efforts are that the longer the follow-up periods are, the more likely the treatments evaluated will have been improved or even superseded by new ones. Hence, the apparent limitation of the present study—its open-label structure—may in fact be an advantage for the reasons just given. Sophisticated machine learning methods will be essential for increasing confidence in adducing cause-effect relationships between training and BrainHealth Index in such an approach.

## Conclusions

In summary, the primary contribution of this pilot study was the development and online testing of the first composite BrainHealth Index to measure and monitor brain health in a holistic framework across the adult lifespan. The key goal of the BrainHealth Index was not to detect or diagnose problems, but rather to motivate individuals to take charge of adopting healthy habits to elevate their brain performance, regardless of the level at which they started. Our findings provide promising evidence that people found the information gleaned from the personalized online BrainHealth Index useful and applicable to their everyday lives. In support of this view, 95% of the individuals signed up to participate in the BrainHealth Project for the next 10 years. Moreover, with greater access to brain health literacy and tactical brain strategies to deploy this information, healthy adults took steps to be proactive about their brain health.

We recognize that the findings yield at least as many questions as they answer. It is important to note that these positive brain health gains were achieved at a time when individuals were burdened with dramatic life changes due to the pandemic. The results offer preliminary evidence to support the perspective that taking time to focus on building brain capacity and resilience during tough times may be especially relevant, instead of waiting until life returns to a more “normal state,” a better choice is perhaps to intercept problems before they worsen. This possibility warrants careful attention. We suggest that resilience is only possible with brain health.

Incorporating machine learning and other sophisticated data analytic methods into the large comprehensive data over the lifespan will help explore the vast opportunities to apply the science of neuroplasticity to unlock human potential. Promoting brain health entails making the most of the brain's capacity to thrive in different contexts and involves strengthening capacities rather than simply remedying deficits when they manifest themselves. This pilot study paves the way for larger-scale efforts to determine whether monitoring and promoting brain health will achieve greater lifelong capacities to build resilient brain systems that respond to life's unknowns and constant changes while averting decline by fine-tuning the brain's complex highly synchronized circuits.

## Data Availability Statement

The raw data supporting the conclusions of this article will be made available by the authors, without undue reservation.

## Ethics Statement

The studies involving human participants were reviewed and approved by University of Texas at Dallas IRB. The patients/participants provided their written informed consent to participate in this study.

## Author Contributions

SC designed the study, interpreted the results, and wrote the manuscript. JF assisted with study design, directed study operations, assisted with development of the online platform, contributed to the manuscript, and edited manuscript. IR contributed content the online training modules, advised on some of the measures comprising the BrainHealth Index, interpreted the results, and wrote the manuscript. MD'E revised the manuscript. GL assisted with study design. JZ performed oversight of training delivery design and execution, collected and scored data, and contributed to manuscript. SV supervised coaching, collected and scored data, and contributed to manuscript. EV designed training protocol, collected and scored data, assisted with the literature review, and contributed to the manuscript. LC collected and scored data, assisted with the literature review, and reviewing of the manuscript. AT supervised and contributed to the development of the online training platform and training modules. JS performed analyses, interpreted results, wrote and edited the manuscript. All authors contributed to the article and approved the submitted version.

## Conflict of Interest

The authors declare that the research was conducted in the absence of any commercial or financial relationships that could be construed as a potential conflict of interest.
